# Diabetes, insulin use and Helicobacter pylori eradication: a retrospective cohort study

**DOI:** 10.1186/1471-230X-12-46

**Published:** 2012-05-09

**Authors:** Chin-Hsiao Tseng

**Affiliations:** 1 Department of Internal Medicine, National Taiwan University Hospital, No. 7 Chung-Shan South Road, Taipei, Taiwan; 2 2Department of Internal Medicine, National Taiwan University College of Medicine, Taipei, Taiwan

**Keywords:** Diabetes, Helicobacter pylori, Insulin, Gastric cancer, National Health Insurance, Taiwan

## Abstract

**Background:**

Diabetic patients may have a higher risk of gastric cancer. However, whether they have a higher incidence of Helicobacter pylori (HP) eradication is not known. Furthermore, whether insulin use in patients with type 2 diabetes may be associated with a higher incidence of HP eradication has not been investigated.

**Methods:**

This is a retrospective cohort study. The reimbursement databases from 1996 to 2005 of 1 million insurants of the National Health Insurance in Taiwan were retrieved. After excluding those aged <25 years, cases of gastric cancer, cases receiving HP eradication before 2005, patients with type 1 diabetes mellitus and those with unknown living region, the reimbursement data of a total of 601,441 insurants were analyzed. Diabetes status and insulin use in patients with type 2 diabetes before 2005 were the main exposures of interest and the first event of HP eradication in 2005 was the main outcome evaluated. HP eradication was defined as a combination use of proton pump inhibitor or H2 receptor blockers, plus clarithromycin or metronidazole, plus amoxicillin or tetracycline, with or without bismuth, in the same prescription for 7-14 days. The association between type 2 diabetes/insulin use and HP eradication was evaluated by logistic regression, considering the confounding effect of diabetes duration, comorbidities, medications and panendoscopic examination.

**Results:**

In 2005, there were 10,051 incident cases receiving HP eradication. HP eradication was significantly increased with age, male sex, diabetes status, insulin use, use of calcium channel blocker, panendoscopic examination, hypertension, dyslipidemia, chronic obstructive pulmonary disease, stroke, nephropathy, ischemic heart disease and peripheral arterial disease. Significant differences were also seen for occupation and living region. Medications including statin, fibrate, angiotensin-converting enzyme inhibitor/angiotensin receptor blocker and oral anti-diabetic agents were not associated with HP eradication. The adjusted odds ratios for diabetes, insulin use and use of calcium channel blocker was 1.133 (1.074, 1.195), 1.414 (1.228, 1.629) and 1.147 (1.074, 1.225), respectively.

**Conclusions:**

Type 2 diabetes and insulin use in the diabetic patients are significantly associated with a higher incidence of HP eradication. Additionally, use of calcium channel blocker also shows a significant association with HP eradication.

## Background

Our population-based cohort study showed that patients with type 2 diabetes mellitus (T2DM) have a significantly higher risk of gastric cancer mortality [1]. Helicobacter pylori (HP) infection is the most important etiology for gastric cancer, and its eradication can significantly reduce gastric cancer in carriers without precancerous lesions [2].

However, the link between diabetes and HP infection has been inconsistently reported. Case-control studies suggested that patients with type 1 diabetes mellitus (T1DM) do not have a higher prevalence of HP infection [3,4] and the prevalence may decrease with longer duration of diabetes [3]. Successful HP eradication rates in patients with T1DM and T2DM are 62% and 50%, respectively, which are much lower than the recommended 80% [5-7]. Furthermore, reinfection rate is higher in patients with T1DM [8,9], and it may deteriorate metabolic control, leading to the requirement of higher insulin dosage and development of diabetic complications [8,9].

Studies regarding HP infection rate in patients with T2DM are still scarce. A hospital-based case-control study from Pakistan enrolling 74 patients with T2DM and 74 non-diabetic controls suggested that diabetic patients have a higher infection rate (73% vs. 51.4%) [10]. Similarly, a higher infection rate is observed in 210 patients with T2DM (vs. 210 controls) in a study from the United Arab Emirates [11]. On the other hand, a Turkish study in 141 patients with T2DM and 142 controls showed no significant difference between the two groups [12].

Based on the above observations, it is reasonable to hypothesize that diabetic patients might have a higher HP infection rate or a higher clinical activity of the infection requiring eradication therapy. It is also possible that patients with T2DM and with poor glycemic control, who require insulin therapy, may represent a group of high risk patients for HP eradication. Population-based study and incidence data evaluating these hypotheses have not been reported. Therefore, by using the population-based reimbursement databases of the National Health Insurance (NHI) in Taiwan, the present study tests these hypotheses by evaluating the incidence and odds ratios of HP eradication in patients with T2DM versus non-diabetic subjects; and in insulin users versus non-users among the patients with T2DM. The effects of diabetes duration, comorbidities, medications and the frequency of panendoscopic examination (PES) were also considered in the analyses.

## Methods

### Study population

This is a retrospective cohort study using the reimbursement databases of the NHI of Taiwan. According to the Ministry of Interior, >98.0% of the Taiwanese population in 2005 (22,770,383: 11,562,440 men and 11,207,943 women) were covered by the NHI [13]. A random sample of 1,000,000 insurants in 2005 was created by the National Health Research Institute. The National Health Research Institute is the only institute approved, as per local regulations, for conducting sampling of a representative sample of the whole population for the year 2005 with a predetermined sample size of 1,000,000 individuals. The reimbursement databases of these individuals were retrieved and could be provided for academic research after approval. The identification information was scrambled for the protection of the privacy of the individuals.

**Figure 1:**
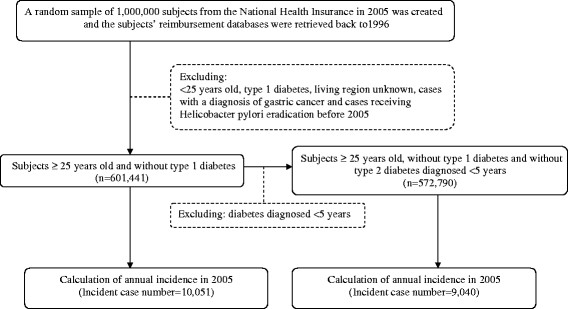
Flowchart showing the procedures in the calculation of the annual incidence of Helicobacter pylori eradication in 2005 in Taiwan using the National Health Insurance database.

Figure 1 shows a flowchart for selecting cases for the study. After excluding subjects <25 years old, patients with T1DM (in Taiwan, patients with T1DM were issued a “Severe Morbidity Card” after certified diagnosis), living region unknown, cases with a diagnosis of gastric cancer and cases receiving HP eradication before 2005, the data of 601,441 subjects were analyzed.

### Data retrieved from NHI databases

The reimbursement databases were available back to 1996. Identification number, sex, birth date, medications and diagnostic codes based on the *International Classification of Diseases, Ninth Revision, Clinical Modification* (ICD-9-CM) were retrieved. Diabetes was coded 250.1-250.9 and gastric cancer 151. The comorbidities (ICD-9-CM codes) included hypertension (401-405), chronic obstructive pulmonary disease (490-496, a surrogate for smoking), stroke (430-438), nephropathy (580-589), ischemic heart disease (410-414), peripheral arterial disease (250.7, 785.4, 443.81, 440-448), eye disease (250.5, 362.0, 369, 366.41, 365.44), obesity (278) and dyslipidemia (272.0-272.4).

Medications included statin, fibrate, angiotensin-converting enzyme inhibitor and/or angiotensin receptor blocker, calcium channel blocker, sulfonylurea, metformin, insulin, acarbose, pioglitazone and rosiglitazone. The NHI insurants were classified according to occupation and this served as a surrogate for socioeconomic status. The living region served as a surrogate for geographical distribution of some environmental exposure. Occupation was categorized as I: civil servants, teachers, employees of governmental or private business, professionals and technicians; II: people without particular employers, self-employed or seamen, III: farmers or fishermen; and IV: low-income families supported by social welfare or veterans. Living region was categorized as Taipei, Northern, Central, Southern and Kao-Ping/Eastern.

The methods for identifying patients receiving HP eradication therapy in a previous study were followed [14]. The details for all therapeutic regimens are shown in the supplementary Table 1 of this earlier paper. In brief, HP eradication therapy was defined as a combination use of proton pump inhibitor or H2 receptor blockers, plus clarithromycin or metronidazole, plus amoxicillin or tetracycline, with or without bismuth, in the same prescription order for 7-14 days.

**Table 1:** Baseline characteristics of study subjects aged 25 years or older by diabetes status

**Variable**	**Diabetes mellitus**				
	**No**		**Yes**		***P***** value**
	***n***** or mean**	**% or SD**	***n*****or mean**	**% or SD**	
*n* (%)	511519	85.05	89922	14.95	
Age (years)	43.98	14.00	58.01	14.95	<0.0001
Sex (men, %)	253626	49.58	39950	44.43	<0.0001
Hypertension (%)	79292	15.50	50549	56.21	<0.0001
Chronic obstructive pulmonary disease (%)	105693	20.66	36273	40.34	<0.0001
Stroke (%)	24744	4.84	18484	20.56	<0.0001
Nephropathy (%)	24780	4.84	15971	17.76	<0.0001
Ischemic heart disease (%)	40465	7.91	28523	31.72	<0.0001
Peripheral arterial disease (%)	15732	3.08	12683	14.10	<0.0001
Eye disease (%)	1170	0.23	8763	9.75	<0.0001
Obesity (%)	4180	0.82	2410	2.68	<0.0001
Dyslipidemia (%)	51126	9.99	44973	50.01	<0.0001
Statin (%)	19608	3.83	24481	27.22	<0.0001
Fibrate (%)	16084	3.14	19053	21.19	<0.0001
Angiotensin-converting enzyme inhibitor/ Angiotensin receptor blocker (%)	6904	1.35	6638	7.38	<0.0001
Calcium channel blocker (%)	34889	6.82	18908	21.03	<0.0001
Occupation (%)					
I	282985	55.32	35103	39.04	<0.0001
II	82588	16.15	17037	18.95	
III	61580	12.04	19708	21.92	
IV	84366	16.49	18074	20.10	
Living region (%)					
Taipei	193494	37.83	31534	35.07	<0.0001
Northern	74045	14.48	11182	12.44	
Central	91087	17.81	15664	17.42	
Southern	66785	13.06	14807	16.47	
Kao-Ping/Eastern	86108	16.83	16735	18.61	
Panendoscopic examination in 2005	8211	1.61	2834	3.15	<0.0001
Panendoscopic examination in 1997-2005	37759	7.38	11762	13.08	<0.0001
**Diabetic patients only**					
Sulfonylurea (%)	-	-	37211	41.38	
Metformin (%)	-	-	33106	36.82	
Insulin (%)	-	-	6010	6.68	
Acarbose (%)	-	-	6968	7.75	
Pioglitazone (%)	-	-	2329	2.59	
Rosiglitazone (%)	-	-	6538	7.27	

Data are expressed as *n* (%) or mean (SD). *Occupation categories are explained in “Materials and Methods”.

### Statistical analyses

Diabetes status and insulin use were considered as the exposures of interest and HP eradication as the outcome in the study. To assure the correctness of temporal order of exposure and outcome, age, diabetes status, diabetes duration, use of insulin or other medications, and comorbidities were recorded as a status or diagnosis before January 1, 2005; and HP eradication was recorded as a first event occurring in the year 2005. PES done in 2005 and from 1997 to 2005 were both analyzed. The purpose to include PES done in 2005 was to assure the temporal proximity of performing PES which might be related to the conduction of HP eradication.

The baseline characteristics between diabetic and non-diabetic subjects were compared by Student’s *t* test for continuous variables and by Chi square test for categorical variables.

Chi square test examined the differences of annual incidence of HP eradication among subgroups of age (25-44, 45-54, 55-64 and ≥65 years), sex, diabetes (for all subjects), insulin use (for diabetic patients only), occupation and living region, for all subjects and for the diabetic patients only separately, before and after excluding patients with diabetes diagnosed <5 years (Figure 1).

Logistic regression estimated the mutually-adjusted odds ratios for HP eradication in all subjects and in the diabetic patients only. For the diabetic patients only, the independent variables included age, sex, PES (1997-2005), the above-mentioned comorbidities, living region, occupation and all the above-mentioned medications. For all subjects, the independent variables included age, sex, diabetes, PES (1997-2005), the above-mentioned comorbidities, medications other than anti-diabetic therapies (i.e., statin, fibrate, angiotensin-converting enzyme inhibitor and/or angiotensin receptor blocker, calcium channel blocker), living region and occupation.

Additional logistic models were created to examine whether the magnitude of the odds ratios for diabetes status (for all subjects), diabetes duration (for all subjects) and insulin use (for the diabetic patients only) might change by including different sets of covariates. Model I was adjusted for age and sex; model II for age, sex, occupation and living region; model III for age, sex, occupation, living region, PES (in 2005), hypertension, chronic obstructive pulmonary disease, stroke, nephropathy, ischemic heart disease, peripheral arterial disease, eye disease, obesity and dyslipidemia; model IV for age, sex, occupation, living region, PES (in 2005), hypertension, chronic obstructive pulmonary disease, stroke, nephropathy, ischemic heart disease, peripheral arterial disease, eye disease, obesity, dyslipidemia, statin, fibrate, angiotensin converting enzyme inhibitor/angiotensin receptor blocker and calcium channel blocker (model IV in diabetic patients only was additionally adjusted for oral anti-diabetic agents including sulfonylurea, metformin, acarbose, pioglitazone and rosiglitazone).

Analyses were conducted using SAS statistical software, version 9.1 (SAS Institute, Cary, NC). Data were expressed as mean (standard deviation) for continuous variables or number (%) for categorical variables. *P* < 0.05 was considered statistically significant.

## Results

Table 1 compares the baseline characteristics between diabetic and non-diabetic subjects. The diabetic patients were older and female predominant, and had higher prevalence rates of comorbidities, medication use and PES (in 2005 or 1997-2005).

The annual incidences of HP eradication in different groups are shown in Table 2. HP eradication rates increased with increasing age, and were higher in men, diabetic patients and insulin users. Significant differences were also seen for occupation and living region.

**Table 2:** Annual incidence (per 100,000) of Helicobacter pylori eradication in 2005 in Taiwan

**Variable**	**Excluding diabetes diagnosed <5 years**					
		**No**			**Yes**	
	*n*	**Incidence**	***P***	***n***	**Incidence**	***P***
**All subjects**						
Age (years)						
25-44	3250	1009.44	<0.0001	3066	974.82	<0.0001
45-54	2370	1805.69		2134	1725.63	
55-64	1687	2469.52		1462	2352.68	
≥65	2744	3433.69		2378	3281.67	
Sex						
Men	5142	1751.51	<0.0001	4612	1648.96	<0.0001
Women	4909	1594.53		4428	1510.76	
Diabetes						
No	7129	1393.69	<0.0001	7129	1393.69	<0.0001
Yes	2922	3249.48		1911	3118.93	
Occupation*						
I	4272	1343.02	<0.0001	3915	1277.81	<0.0001
II	1815	1821.83		1640	1744.48	
III	2023	2488.68		1764	2332.50	
IV	1941	1894.77		1721	1778.46	
Living region						
Taipei	3471	1542.47	<0.0001	3123	1455.34	<0.0001
Northern	1220	1431.47		1103	1349.10	
Central	1670	1564.39		1486	1461.00	
Southern	1623	1989.17		1449	1874.95	
Kao-Ping/Eastern	2067	2009.86		1879	1928.17	
**Diabetic patients only**						
Age (years)						
25-44	374	1999.57	<0.0001	190	1687.09	<0.0001
45-54	568	2789.51		332	2598.83	
55-64	677	3435.33		452	3339.24	
≥65	1303	4183.12		937	3953.92	
Sex						
Men	1380	3454.32	0.0020	850	3260.95	0.0818
Women	1542	3085.73		1061	3013.78	
Insulin use						
No	2619	3121.13	<0.0001	1678	2979.72	<0.0001
Yes	303	5041.60		233	4700.42	
Occupation*						
I	963	2743.36	<0.0001	606	2589.96	<0.0001
II	521	3058.05		346	3028.98	
III	778	3947.64		519	3694.74	
IV	660	3651.65		440	3547.53	
Living region						
Taipei	962	3050.68	0.0002	614	2910.64	<0.0001
Northern	330	2951.17		213	2761.57	
Central	489	3121.81		305	2870.86	
Southern	548	3700.95		374	3562.92	
Kao-Ping/Eastern	593	3543.47		405	3570.80	

*n* = case number of Helicobacter pylori eradication.

*Occupation categories are explained in “Materials and Methods”.

Chi-square test was used for the statistical analysis.

The mutually-adjusted odds ratios for HP eradication are shown in Table 3. For all subjects, age, male sex, diabetes, PES (1997-2005), hypertension, chronic obstructive pulmonary disease, stroke, nephropathy, ischemic heart disease, peripheral arterial disease, dyslipidemia, calcium channel blocker, living region (northern and central) and occupation (III and IV) were significantly associated with a higher incidence. Except for dyslipidemia and living region in northern Taiwan, the significant variables for the diabetic patients only were all the same as in the model for all subjects. Furthermore, insulin use was significantly associated with HP eradication.

**Table 3:** Mutually-adjusted odds ratios for Helicobacter pylori eradication in 2005 in Taiwan

**Study/Variable**	**Interpretation**	**Adjusted odds ratio (95% confidence interval)**	
		**All subjects**	**Diabetic patients only**
Age	Every 1-year increment	1.014 (1.012, 1.015)	1.008 (1.005, 1.011)
Sex	Men vs. Women	1.220 (1.171, 1.270)	1.155 (1.071, 1.247)
Diabetes	Yes vs. No	1.133 (1.074, 1.195)	--
Panendoscope examination (1997-2005)	Yes vs. No	8.940 (8.573, 9.321)	5.592 (5.173, 6.045)
Hypertension	Yes vs. No	1.199 (1.129, 1.273)	1.215 (1.098, 1.345)
Chronic obstructive pulmonary disease	Yes vs. No	1.257 (1.202, 1.314)	1.214 (1.121, 1.313)
Stroke	Yes vs. No	1.119 (1.051, 1.191)	1.096 (1.000, 1.201)
Nephropathy	Yes vs. No	1.268 (1.193, 1.347)	1.314 (1.203, 1.435)
Ischemic heart disease	Yes vs. No	1.246 (1.179, 1.317)	1.183 (1.086, 1.289)
Peripheral arterial disease	Yes vs. No	1.126 (1.048, 1.211)	1.133 (1.025, 1.252)
Eye disease	Yes vs. No	0.937 (0.833, 1.053)	0.898 (0.784, 1.029)
Obesity	Yes vs. No	0.971 (0.816, 1.155)	0.815 (0.627, 1.061)
Dyslipidemia	Yes vs. No	1.174 (1.110, 1.242)	1.034 (0.948, 1.127)
Statin	Yes vs. No	1.013 (0.946, 1.086)	1.015 (0.923, 1.116)
Fibrate	Yes vs. No	1.015 (0.944, 1.091)	1.007 (0.913, 1.110)
Angiotensin-converting enzyme inhibitor/ Angiotensin receptor blocker	Yes vs. No	1.011 (0.912, 1.121)	1.051 (0.913, 1.211)
Calcium channel blocker	Yes vs. No	1.147 (1.074, 1.225)	1.108 (1.004, 1.224)
Living region	Northern vs. Taipei	1.103 (1.042, 1.168)	1.082 (0.968, 1.208)
	Central vs. Taipei	1.124 (1.056, 1.195)	1.140 (1.022, 1.271)
	Southern vs. Taipei	1.027 (0.969, 1.088)	1.047 (0.941, 1.165)
	Kao-Ping/Eastern vs. Taipei	0.988 (0.924, 1.058)	0.979 (0.859, 1.116)
Occupation	II vs. I	1.022 (0.961, 1.087)	1.023 (0.910, 1.148)
	III vs. I	1.317 (1.235, 1.404)	1.286 (1.144, 1.445)
	IV vs. I	1.308 (1.235, 1.386)	1.205 (1.080, 1.345)
Sulfonylurea	Yes vs. No	-	0.978 (0.875, 1.093)
Metformin	Yes vs. No	-	0.972 (0.867, 1.091)
Insulin	Yes vs. No	-	1.414 (1.228, 1.629)
Acarbose	Yes vs. No	-	1.004 (0.863, 1.167)
Pioglitazone	Yes vs. No	-	0.999 (0.787, 1.269)
Rosiglitazone	Yes vs. No	-	0.939 (0.800, 1.101)

*Occupation categories are explained in “Materials and Methods”.

The unadjusted and adjusted odds ratios for HP eradication for diabetes status (for all subjects), diabetes duration (for all subjects) and insulin use (for diabetic patients only) are shown in Table 4. Diabetes was associated with a significantly higher odds ratio of HP eradication in all models, though the odds ratio attenuated when more covariates were adjusted, especially when the comorbidities were considered (models III and IV). The odds ratios attenuated with increasing diabetes duration and with adjustment for covariates, especially when comorbidities and medications were included. The odds ratios became insignificant for diabetes duration of more than 3 years in models III and IV. Insulin use was significantly associated with a higher odds ratio though the magnitude of the odds ratios attenuated slightly after adjustment for covariates.

**Table 4:** Odds ratios for Helicobacter pylori eradication for diabetes status, diabetes duration and insulin use

**Diabetes-related variable**	**Odds ratio (95% confidence interval)**				
	**Unadjusted**		**Adjusted**		
		**Model I**	**Model II**	**Model III**	**Model IV**
**All subjects**					
Non-diabetes	1.000	1.000	1.000	1.000	1.000
Diabetes	2.376 (2.275, 2.482)	1.644 (1.569, 1.723)	1.633 (1.558, 1.711)	1.132 (1.062, 1.208)	1.137 (1.065, 1.213)
**All subjects**					
Non-diabetes	1.000	1.000	1.000	1.000	1.000
Diabetes <1 year	3.596 (3.217, 4.020)	2.736 (2.444, 3.061)	2.721 (2.431, 3.045)	1.772 (1.522, 2.062)	1.772 (1.522, 2.062)
Diabetes 1-3 years	2.273 (2.062, 2.506)	1.695 (1.536, 1.871)	1.681 (1.523, 1.855)	1.293 (1.139 1.468)	1.289 (1.135, 1.463)
Diabetes 3-5 years	2.178 (1.977, 2.398)	1.587 (1.439, 1.750)	1.575 (1.428, 1.737)		1.053 (0.927, 1.196)
Diabetes ≥5 years	2.303 (2.182, 2.430)	1.513 (1.428, 1.602)	1.503 (1.419, 1.592)		1.022 (0.944, 1.106)
**Diabetic patients only**					
Non-insulin users	1.000	1.000	1.000	1.000	1.000
Insulin users	1.648 (1.459, 1.862)	1.495 (1.322, 1.690)	1.483 (1.311, 1.677)	1.319 (1.127, 1.543)	1.389 (1.178, 1.639)*

I: Adjusted for age and sex.

II: Adjusted for age, sex, occupation and living region.

III: Adjusted for age, sex, occupation, living region, panendoscopic examination (in 2005), hypertension, chronic obstructive pulmonary disease, stroke, nephropathy, ischemic heart disease, peripheral arterial disease, eye disease, obesity and dyslipidemia.

IV: Adjusted for age, sex, occupation, living region, panendoscopic examination (in 2005), hypertension, chronic obstructive pulmonary disease, stroke, nephropathy, ischemic heart disease, peripheral arterial disease, eye disease, obesity, dyslipidemia, statin, fibrate, angiotensin converting enzyme inhibitor/angiotensin receptor blocker and calcium channel blocker.

*Model IV in diabetic patients only was additionally adjusted for oral anti-diabetic agents including sulfonylurea, metformin, acarbose, pioglitazone and rosiglitazone.

## Discussion

This study provided for the first time a nation-wide population-based analysis with a large sample size on the association between T2DM and HP eradication. Diabetes was significantly associated with a higher incidence of HP eradication (Tables 2, 3, 4), independent of comorbidities, medications, PES, occupation and living region (Tables 3 and 4). The odds ratios attenuated with increasing diabetes duration, probably due to the increased occurrence of comorbidities, which might also affect the incidence of HP eradication (Table 4). Furthermore, use of calcium channel blockers (Table 3) and insulin use in the diabetic patients (Tables 2, 3, 4) were also significantly associated with HP eradication.

The higher incidence of HP eradication associated with diabetes could be explained by the following possibilities: the diabetic patients might have a higher chance of being detected for HP infection, or they might have a higher HP infection rate or a higher activity of HP infection. A higher detection rate is possible because the diabetic patients were more prone to have PES (Table 1). However, this could not explain the whole picture because the higher incidence of HP eradication associated with diabetes remained significant after adjustment for PES and other covariates (Tables 3 and 4). Currently we do not have HP infection rates in Taiwanese diabetic and non-diabetic subjects. Some studies suggested that the HP infection rates are similar between non-diabetic and diabetic subjects in patients with either T1DM [3,4] or T2DM [12]. If this is the case, the higher incidence of HP eradication associated with diabetes (Tables 2,3,4) may implicate that HP infection in the diabetic patients is more clinically active with more severe symptoms, leading to its diagnosis and the use of medications for its eradication.

Insulin use was consistently associated with a higher incidence of HP eradication, but none of the oral anti-diabetic agents was (Tables 2,3,4). Insulin use may be a proxy for uncontrollable hyperglycemia with more severe disease. Therefore this observation might be explained in the following ways. HP infected patients might have deteriorated metabolic control [8,9] and required insulin therapy; or they might have more severe diabetic conditions (e.g., diabetic gastroparesis) with gastrointestinal symptoms leading to more aggressive examination and diagnosis of HP infection.

A significantly higher rate of HP eradication was seen in subjects taking calcium channel blockers (Table 3). It is interesting that calcium channel blockers are also associated with gastroesophageal reflux disease, probably due to its relaxation effect on lower esophageal sphincter [15]. HP infection can induce pepsinogen release from chief cells (which may induce and aggravate peptic ulcer disease) via mechanisms involving calcium and calmodulin [16]. However, L-type calcium channel is not responsible for the pepsinogen release induced by HP [16]. Therefore it is unlikely that calcium channel blockers used in clinical practice would affect the peptic ulcers induced by HP. It was possible that the gastrointestinal symptoms associated with its use that led to the diagnosis of HP infection.

Recent studies suggest that HP eradication may reverse atrophic gastritis and improve intestinal metaplasia, which may contribute to the reduction of gastric cancer occurrence [17-19]. Therefore, early diagnosis of HP infection with clinical use of medications to eradicate the infection is not only important for the treatment of the clinical symptoms related to the infection, but also for the prevention of gastric cancer. One clinical implication of the present study is that patients with T2DM, especially those treated with insulin, may belong to a high risk group requiring special medical attention.

HP infection rate in a community-based study in Taiwan was 54.4%, and it showed age-dependency without sexual difference [20]. Although the present study suggested that clinical presentation of HP eradication was age-dependent (Tables 2 and 3), it also demonstrated a higher incidence of HP eradication in the male population (Tables 2 and 3). The reasons for a higher risk of HP eradication in men are still unknown. One possibility is that the activity of HP infection could be higher in men, which is correspondent to the higher risk of gastric cancer in men than in women in Taiwan [1].

Except for eye disease, obesity and dyslipidemia (in diabetic patients only) all other comorbidities were significantly associated with HP eradication (Table 3). This observation explained the attenuated odds ratios with prolonged diabetes duration (Table 4), when chronic complications might set in and interfere with the association between diabetes and HP eradication.

Some studies suggested that people living in crowded condition or with lower socioeconomic status may have a higher risk of HP infection [21-23]. In Taiwan, Taipei City is the most populated area. However, residents in relatively sparse area of Central Taiwan had significantly higher incidence of HP eradication than people living in other regions (Table 3). This suggested that living in crowded condition might not be an important predisposing factor. On the other hand, people with an occupation as farmers or fishermen (occupation III) or with low family income (occupation IV) consistently showed significantly higher odds ratios (Table 3), suggesting a possible role of socioeconomic status or its related condition of hygiene.

This study has several strengths. It is population-based with a large nationally representative sample, therefore, the study is not likely to be biased with respect to diabetes status or records of HP eradication. Because NHI is a universal and mandatory insurance with very high coverage but low co-payments, the detection rate would not tend to differ among different social classes.

Limitations included a lack of actual measurement of some recognized confounders such as personal hygiene, living condition, blood groups and genetic factors. We also did not have biochemical data including blood glucose, hemoglobin A_1c_ and lipid profiles to evaluate whether HP infection can affect metabolic control. This study evaluated the incidence of HP eradication and not the prevalence or incidence of HP infection. However, because most people infected with HP do not develop clinical disease [21], estimation of prevalence or incidence of HP infection may not have clinical relevance as the evaluation of HP eradication does. Another concern is that there might be considerable under-diagnosis and under-treatment of HP infection. However, if the misclassification of the outcome is non-differential, an underestimation of the odds ratios is expected.

## Conclusions

This study shows a significantly higher incidence of HP eradication in patients with T2DM and in insulin users among the diabetic patients. The odds ratios attenuated with increasing diabetes duration, probably due to the occurrence of other comorbidities. Additionally, lower socioeconomic status and use of calcium channel blockers consistently show a higher rate of HP eradication, but the uses of oral anti-diabetic agents do not. The underlying causes for the link between T2DM and the use of medications and HP eradication are clinically important but require further investigation.

## Abbreviations

HP = Helicobacter pylori; ICD-9-CM = International Classification of Diseases, Ninth Revision, Clinical Modification; NHI = National Health Insurance; PES = Panendoscopic examination; T1DM = Type 1 diabetes mellitus; T2DM = Type 2 diabetes mellitus.

## Competing interests

The author declares that he has no competing interests.

## Author’s contributions

CHT researched data and wrote manuscript.

## Pre-publication history

The pre-publication history for this paper can be accessed here:

http://www.biomedcentral.com/1471-230X/12/46/prepub
